# Do Forms of Silicon Other than Orthosilicic Acid, Application Date and Dose Have a Beneficial Effect on Sugar Beet Yield?

**DOI:** 10.3390/plants15101449

**Published:** 2026-05-09

**Authors:** Arkadiusz Artyszak, Dariusz Gozdowski, Magda Litwińczuk-Bis

**Affiliations:** Institute of Agriculture, Warsaw University of Life Sciences—SGGW, Nowoursynowska 159, 02-787 Warsaw, Poland; dariusz_gozdowski@sggw.edu.pl (D.G.); magdalitwinka@wp.pl (M.L.-B.)

**Keywords:** calcium silicate, micronized silica, silicon form, sodium metasilicate, sugar beet

## Abstract

In 2017–2019, in a field experiment in Sahryń, Poland (50°40′42″ N, 23°47′35″ E), the effect of foliar application of various forms of silicon (calcium silicate—CS, sodium metasilicate—SM, and micronized silica—MS) and the timing of their application (at the 6-leaf stage of sugar beet, 7 and 14 days later) at single and double doses on physiological parameters, yield, and technological quality of sugar beet roots was studied. The silicon form significantly modified all assessed physiological parameters (NDVI, LAI, and PAR absorption) at all measurement dates. The application date had a significant effect on the assessed parameters at later measurement dates, while the product dose had essentially no effect. The silicon form significantly affected root yield and technological quality, with the exception of α-amino nitrogen content, biological sugar yield, and pure sugar yield. Foliar application of CS and SM resulted in the highest root yield, biological sugar yield, and pure sugar yield, as well as the highest sugar content and the lowest Na content in CS roots. The timing of foliar application significantly affected root yield, biological sugar yield, and α-amino N content. The highest root yield (88.53 t ha^−1^) and biological sugar yield (15.47 t ha^−1^) were achieved when the application was performed 14 days after the 6-leaf stage. Simultaneously, the technological quality of the roots deteriorated due to a significant increase in α-amino N content. Application of a double dose of the product significantly increased sugar content and decreased Na content in the roots.

## 1. Introduction

Due to climatic constraints, sugarcane cultivation is impossible in Europe. Consequently, sugar beet in this region is the only raw material for sugar production. The area under sugar beet cultivation in Europe in 2024 was 3.1 million hectares (4.7 million hectares globally), with an average root yield of 62.7 t ha^−1^ (63.1 t ha^−1^ globally), and a harvest of over 197 million tons (over 293 million tons globally) [1]. Climate change is causing plants to be more frequently exposed to unfavorable conditions, which reduce yield and quality. It has been found that the yield of major crops falls by 4.4% for every 1 °C rise in temperature [2]. It has been shown that a 1 °C rise in temperature has reduced global yields of maize by 7.5 ± 5.3%, wheat by 6.0 ± 3.3%, soybean by 6.8 ± 5.9% and rice by 1.2 ± 5.2% [3]. Drought is drastically reducing the quality of agricultural produce [4]. Rising temperatures facilitate the growth of plant pathogens and pests, and also reduce the shelf life of food [5].

In recent years, silicon (Si) has become a key element for improving plant health in difficult conditions, and its use is considered essential for achieving sustainable development in crop production [6]. Silicon modifies the water balance of plants, enhancing their ability to maintain water content under drought conditions by regulating water uptake mechanisms. Furthermore, it reduces plant transpiration not only during drought but also when water supply is adequate [7]. The element modulates the expression of aquaporin genes and the levels of antioxidant enzymes, increasing plants’ adaptive potential to drought [8]. Research findings indicate that silicon aids plants’ adaptation to water deficit by regulating water transport and stabilizing water management in the roots [9]. Silicon also participates in the modification of root cell wall structure, influencing callose deposition and the esterification of pectins and arabinogalactans [10].

It is widely believed that the only form of silicon absorbed by plants is orthosilicic acid, H_4_SiO_4_, and therefore, it is the most effective [11]. At concentrations above 0.1%, silicic acid molecules combine to form polysilicic acid with reduced bioavailability, requiring stabilization, for example, with choline. This increases production costs and, as a result, makes foliar products containing orthosilicic acid expensive [12,13]. Consequently, cheaper products containing silicon in other forms are sought. Numerous experiments demonstrate the beneficial effects of calcium silicate [12,13,14], potassium silicate [13,15,16,17,18], sodium metasilicate [12,13,19,20,21], nanoparticles (SiNPs) [22,23,24,25], and micronized marine calcite [19]. Current research on sugar beet production has failed to clearly determine which form of silicon is most effective in reducing the impact of abiotic and biotic stress factors [12,13]. The same applies to the timing of application [13,16]. Due to the cost of performing applications, efforts are being made to limit their number. Recommendations for the use of individual foliar products include varying doses. Therefore, for agricultural practice, it is important to know whether increasing the manufacturer’s recommended doses is advisable. It is therefore worth investigating the choice of silicon form, the timing of application, the product dosage and the interaction between these factors, as the results of such studies have not yet been published. The aim of the study was to assess the effect of a single foliar application of various forms of silicon (except orthosilicic acid) at various times and doses on plant physiological parameters, yield, and technological quality of sugar beet roots. It was hypothesized that foliar application of products with different compositions and doses has a significant impact on selected plant physiological parameters, yield, and technological quality of sugar beet roots.

## 2. Results

### 2.1. Physiological Parameters of Plants

The assessed physiological parameters of sugar beet plants (NDVI, LAI, and photosynthetically active radiation (PAR) absorption) at each measurement date were significantly influenced by the study year, silicon form, the interaction of study year and silicon form, the interaction of silicon form and application date, and the interaction of study year, silicon form, and application date (Table 1). The interaction of study year and application date was similar, with the exception of NDVI at measurement date 2. In the remaining cases, the influence of the studied factors and their interaction was significant for some physiological parameters and at some measurement dates.

**Figure 1:**
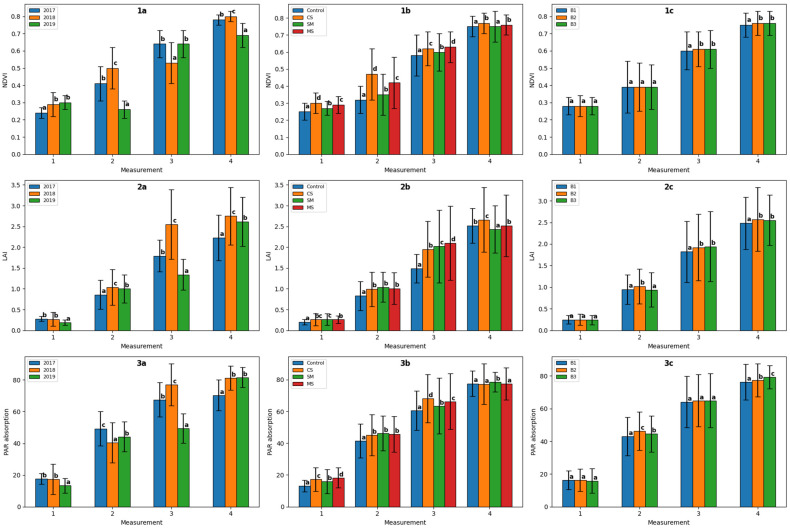
Changes in NDVI (charts **1a**–**1c**), LAI (charts **2a**–**2c**) and PAR absorption (charts **3a**–**3c**) depending on study years (2017, 2018 and 2019—charts **1a**,**2a**,**3a**), silicon form (Control–without Si fertilization, CS: silicon in the form of calcium silicate (Barrier Si-Ca); SM: silicon in the form of sodium metasilicate (ProHorti Si Max); MS: silicon in the form of micronized silica (TopSi)—**1b**,**2b**,**3b**), and application timing (B1—6-leaf stage of sugar beet—BBCH 16; B2—7 days later; B3—14 days later—**1c**,**2c**,**3c**). Vertical lines indicate standard deviations. The same letters indicate no significant differences at *p* = 0.05.

The NDVI value at each measurement date varied significantly across the study years (Figure 1 (1a)). The highest values were recorded on dates 2 and 4 in 2018, on date 1 in 2018 and 2019, and on date 3 in 2017 and 2019. The NDVI value increased in successive measurements. The largest increase was recorded in 2017, and the smallest two years later.

In the years 2017–2019, on the first and second measurement dates, the highest NDVI value was recorded for the combination with CS, on the third date with MS, and on the fourth date with CS and MS (Figure 1(1b)). At the second measurement date, the greatest increase in NDVI values was recorded following the application of CS; however, in subsequent measurements, the differences between the combinations decreased.

For the third and fourth measurement dates, the application on dates B2 and B3 contributed to a significant increase in the NDVI value (Figure 1(1c)).

Dose C2 caused a significant increase in the NDVI value compared to dose C1 only on the third measurement date (Figure S1(1d)).

The LAI value in the first measurement period in 2017 and 2018 was significantly higher than in the third year of the study (Figure 1(2a)). In subsequent measurements, the highest index value was obtained in 2018. This was due to the high amount of rainfall in June 2018.

In the first measurement term, the highest LAI value was found in the combination with SM, in the second term with CS, SM, and MS, in the third term in the combination with MS, and in the fourth term in the combination with CS (Figure 1(2b)). 

Application date B2 allowed for obtaining a significantly higher index value on the second measurement date than on dates B1 and B3 (Figure 1(2c)). However, on the third and fourth measurement dates, the highest LAI value was found on application dates B2 and B3.

The dose of the tested products had no significant effect on the LAI value at any of the measurement dates (Figure S1(2d)).

PAR absorption on the first measurement date was the highest in 2017 and 2018, on the second measurement date in 2017, on the third measurement date in 2018, and on the fourth measurement date in 2018 and 2019 (Figure 1(3a)). This is due to the weather conditions during the measurement period (late May to the second ten days of June), as well as in the preceding period.

All tested silicon forms caused a significant increase in PAR absorption compared to the control combination at all measurement dates, except for the fourth date, when it occurred only for the combination with SM (Figure 1(3b)). On the first date, the highest increase was observed in the combination with MS, on the third date with CS, and on the second date it was similar for all products. It is therefore difficult to say definitively which form of silicon had the most beneficial effect on the PAR value.

PAR absorption on the second and fourth measurement dates on application dates B2 and B3 was significantly higher than on application date B1 (Figure 1(3c)).

In none of the measurement dates did the dose of the product used have a significant effect on the amount of PAR absorption (Figure S1(3d)).

The results presented here demonstrate that weather conditions, and above all the amount of rainfall, prevailing during the measurement period and immediately prior to it, have a significant impact on the physiological parameters assessed. Of the forms of silicon studied, CS had the most beneficial effect on NDVI and LAI. At the time of application, sugar beet plants must have developed a sufficiently large leaf area for the product to be effective.

On average, for the entire study period, the LAI had the highest variability at each measurement date, while the NDVI had the lowest variability, with the exception of the second date (Table 2).

Over the entire study period, a positive relationship was found between root yield and NDVI (on the second and fourth measurement dates), LAI (on the first and third measurement dates), and PAR absorption on the first, second, and third measurement dates (Table 3). In the case of sugar content in roots, a significant relationship was found between NDVI (negative on the second and fourth measurement dates, and positive on the third measurement date), LAI (negative on the first and third measurement dates), and PAR absorption (negative on the first and third measurement dates, and positive on the second measurement date). The relationships between biological sugar yield and pure sugar yield and the assessed physiological parameters were similar. Both traits had a significant and positive relationship with all physiological parameters on each measurement date, except for PAR absorption on the fourth measurement date, when this relationship was negative, and with the NDVI on the first date and the LAI on the second and third dates, when it was insignificant.

### 2.2. Yield and Technological Quality of Sugar Beet Roots

Sugar beet yield and technological quality of roots are significantly influenced by the years of research, the interaction of years of research and silicon form, the interaction of years of research, silicon form and application date, silicon form, and the interaction of silicon form and application date (Table 4).

During the study years, the application of all silicon forms resulted in a significant increase in root yield compared to the control combination (Table 5). Combinations with CS and SM were characterized by significantly higher root yield compared to the MS combination. Application dates B2 and B3 contributed to a significant increase in root yield compared to date B1. The highest root yield was observed in the CS combination applied on date B3 at a dose of C1.

Sugar content in roots in the CS combination was significantly higher, and in the SM and MS combinations significantly lower, than in the control combination (Table 6). Application at B3 resulted in a significant increase in sugar content in roots compared to B2. Roots from the C2 combination had significantly higher sugar content than roots from the C1 combination. The highest sugar content was found in roots from the CS combination applied at B2 and the C1 dose.

The combination with CS was characterized by a significantly higher content of α-amino nitrogen in the roots than the control combination (Table 7). Application dates B1 and B2 contributed to a significant reduction in the content of this molasses-forming component compared to date B3. The lowest α-amino nitrogen content was observed in roots from the combination with MS applied on date B1 at a dose of C2.

All tested forms of silicon caused a significant increase in K content in roots compared to the control combination (Table 8). Application date B1 allowed for a significantly lower content of the tested molasses-forming component in roots compared to date B3. The lowest K content was observed in roots from the control combinations.

The combination with CS was characterized by a significantly lower Na content in roots, and the combinations with SM and MS were characterized by a significantly higher Na content in roots compared to the control combination (Table 9). Application at the B1 date resulted in a significantly lower content of the molasses-forming component compared to application at the B3 date. Roots from the combination with the C2 dose had a significantly lower Na content than combinations with the C1 dose. The lowest Na content was observed in roots from the combination with CS applied at the B1 date and the C1 dose.

The combination with MS was characterized by a significantly higher alkalinity coefficient than the control and CS combinations (Table 10). Application at term B2 resulted in a significant increase in the value of the tested trait compared to term B3. The highest alkalinity coefficient value was observed in the combination with MS applied at term B1 at a dose of C2.

The use of CS contributed to a significant increase in biological sugar yield and pure sugar yield compared to the other combinations, and SM and MS compared to the control combination (Table 11 and Table 12). Application at the B3 date resulted in a significant increase in sugar yield compared to the B1 date. The highest sugar yield was obtained in the combination with CS applied at the B3 date at the C1 dose.

The highest variability was observed in the Na content in roots (CV = 37.83%), and the lowest in the sugar content in roots (CV = 5.99%) (Table 13).

**Figure 2:**
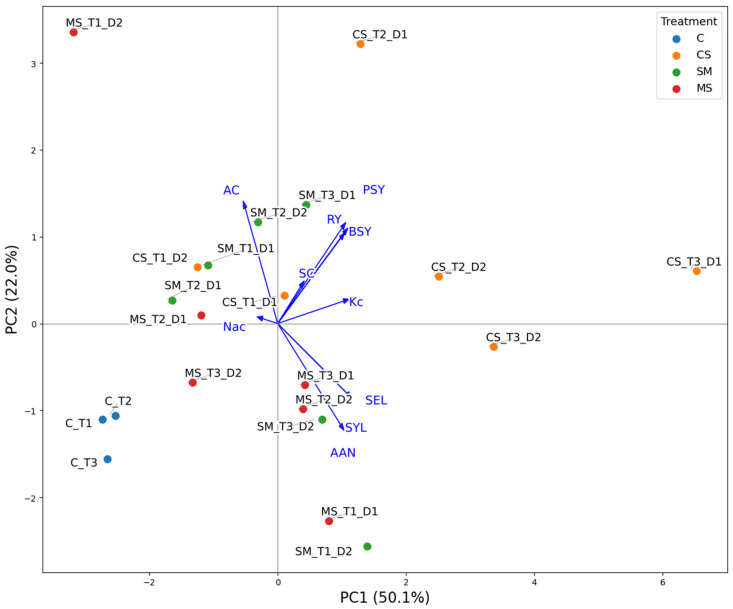
Biplot based on PCA results presenting multivariate relationships between studied variables and multivariate differences between the experimental variants. Rootyield, t ha^−1^ (RY); sugar content, % (SC); potassium content, mmol kg^−1^ (Kc); sodium content, mmol kg^−1^ (Nac); α-amino nitrogen content, mmol kg^−1^ (AAN); biological sugar yield, t ha^−1^ (BSY); standard molasses loss, % (SML); sugar yield losses, % (SYL); alkalinity coefficient (AC); pure sugar yield, t ha^−1^ (PSY).

PCA was performed on the dataset including root yield (RY), sugar content (SC), potassium (Kc), sodium (Nac), α-amino nitrogen (AAN), biological sugar yield (BSY), standard molasses loss (SML), sugar yield losses (SYL), alkalinity coefficient (AC), and pure sugar yield (PSY) (Figure 2). The first two principal components captured the major variation among treatments (in total about 70% of variation). Variable loadings indicate that RY, Kc, BSY, SML, SYL, and PSY are positively correlated, while AAN and AC show negative or independent associations with these traits. Sugar content (SC) and alkalinity (AC) form separate dimensions of variation, highlighting differences in quality characteristics across treatments. Control treatments cluster together with moderate values across all traits. CS treatments (Barrier S-Ca, one or two doses) are more dispersed, some showing high root and sugar yields and others moderate molasses losses and AAN levels. SM treatments (Pro Horti Si Max, one or two doses) are intermediate, varying mainly in sugar losses, AAN, and yield traits. MS treatments (TopSi, one or two doses) show the widest variation, particularly in sugar content, alkalinity, and technological sugar yield. Overall, the PCA highlights clear differences among the treatment series: controls are consistent and moderate, CS treatments tend toward higher yield, SM treatments show moderate quality and yield values, and MS treatments display high variability in both yield and quality traits. Treatments with different doses and application timing are also distinguishable, reflecting the impact of these management factors on the measured characteristics.

Overall, the PCA distinguishes treatments based on their combined yield and quality traits, with C, CS, SM, and MS series showing distinct patterns across the measured variables.

## 3. Discussion

### 3.1. Physiological Parameters

In our research, the physiological parameters of sugar beet plants were significantly influenced by climatic conditions (during the measurement period and also before them), and among the factors studied, by the form of silicon. It is difficult to clearly indicate which form had the most beneficial effect, as the effect varied between measurements dates. Overall, CS proved to be the most effective, with the most positive impact on NDVI and LAI. Of the parameters assessed, the most important seems to be the NDVI, which is used in precision agriculture to assess plant condition, including in studies involving silicon [26]. On the last measurement date, CS and MS had the most beneficial effect on NDVI, LAI on CS, and PAR absorption on SM. Similar results were obtained in other studies [13]. Differentiated soil fertilization with Si, Ca, and Mg and foliar application of CS had a small and ambiguous effect on NDVI values, PAR absorption, and SPAD chlorophyll content in maize leaves. Only in the case of LAI was it found that the vast majority of soil fertilization and foliar fertilization combinations had a significant positive effect on LAI values assessed in the second ten-day period of July [14]. The use of SM in potato production resulted in increased leaf area and LAI [27,28]. In previous studies, the timing of foliar application of products containing silicon in various forms significantly modified some physiological parameters at certain measurement times [13].

### 3.2. Root Yield

In our studies, root yield was significantly influenced by weather conditions during the study years, the form of silicon, and the timing of foliar application. Drought stress is the main factor limiting sugar beet yield in many regions of the world [29]. Studies conducted in Central Europe have shown that soil fertilization with silicon in sugar beet cultivation has a beneficial effect on reducing the effects of drought [30]. Its negative impact can be mitigated by foliar application of silicon, as demonstrated by studies conducted on mung beans [31]. Despite numerous studies, its mechanism of action is still not sufficiently understood. One reason for the improved sugar beet yield may be the increased efficiency of soil phosphorus use [32,33].

Recent research results have confirmed the significant effect of foliar application of SM, OSA and CS [12], and SSN [34] on sugar beet root yield. Combinations with PS, CS, OSA, and SM were characterized by similar root yields [13]. In potato production, a significant increase in tuber yield was observed following foliar application of OSA [35,36]. At the same time, a tendency was observed to increase the share of large tubers in the yield structure [36] or to have no significant effect on tuber yield structure [35,37]. SM application resulted in a significant increase in marketable tuber yield and the share of medium-sized tubers in the marketable yield [38]. However, GMC application contributed to a significant increase in tuber yield and the share of large tubers in the yield [39]. In our own studies, a later application date favored significantly higher root yields. No effect of the application date of various forms of silicon on sugar beet root yield was observed [13]. Foliar application of CS in winter rapeseed significantly (by 8%) increased winter rapeseed seed yield [15]. The combination of soil fertilization with fertilizer containing Si, Ca, and Mg and foliar application of CS resulted in a significant increase in grain yield and dry matter of maize [14]. In maize cultivation, a beneficial effect was obtained by applying OSA in various forms (seed dressing before sowing, spraying the soil before sowing and spraying the plants in the 5–6-leaf stage) [40].

### 3.3. Technological Quality of Roots

When assessing the technological quality of sugar beet roots, the following factors are taken into account: the content of sugar and molasses-forming components (α-amino nitrogen, potassium ions, and sodium). The interrelationships between the molasses-forming components determine the alkalinity coefficient, and the higher the value, the better the technological quality of the raw material.

In our studies, the form of silicon significantly varied the sugar content in roots, which was highest after CS application. Most previous research results indicate a varied effect of foliar-applied silicon products on sugar content in sugar beet roots [12]. In other studies, the application of CS and OSA contributed to a significant increase in sugar content in roots compared to the combination with PS and SM [13]. In a few studies, a significant increase in sugar content in roots was observed as a result of foliar application of SSN [34]. In our own studies, the sugar content in roots varied significantly depending on the application date and was highest at the latest application date. Similar results were obtained in previous studies [13].

Of the molasses-forming components, α-amino nitrogen is considered the most harmful, causing twice as much sugar loss as potassium and sodium ions [41]. In our own studies, the content of this molasses-forming component was significantly modified by the years of study and the application date. Similar α-amino nitrogen content was obtained using CS and SM [12]. Studies with SSN revealed a significant reduction in the content of this component [34], and with OSA, an increase compared to the control combination [12]. The form of silicon (PS, CS, OSA, SM) significantly differentiated the α-amino nitrogen content in other studies [13].

In our studies, the content of this molasses-forming component was highest at the latest application date. No significant effect of the application date on the value of this characteristic was obtained in previous studies [13]. In our own studies, all forms of silicon used caused a significant increase in K content in sugar beet roots compared to the control combination. It is suspected that foliar application of silicon enhances potassium uptake from the soil in sugar beet plants, but the mechanism behind this effect is not yet understood.

Similar results were previously obtained using OSA and CS, while no significant differences were observed in the combination with SM compared to the control combination [12]. A significant increase in K content in roots after CS, OSA, and SM application compared to the combination with PS was observed in other studies [13]. The use of SSN resulted, depending on the variant, in either no change in K content in roots or a significant reduction [34]. In our studies, application at the latest date contributed to obtaining roots with the highest K content. Opposite results were obtained in earlier studies [12].

Na is the least harmful molasses-forming nutrient, which is due, among other things, to its lowest content in roots. In our own studies, CS application contributed to a significant reduction, while SM and MS contributed to an increase in Na content in roots compared to the control combination. Previous research results indicate a varied effect of silicon forms on Na content in sugar beet roots. Application of PS, CS, and SM caused a significant increase in Na content in roots compared to the combination with OSA [13]. Some authors [12] demonstrated a significant increase in Na content in roots after foliar application of OSA, but no significant differences after application of SM and CS. However, application of SSN caused a significant reduction in the content of this molasses-forming nutrient [34].

In our studies, application at the latest date contributed to obtaining roots with the highest Na content. Previous studies found no significant differences in this nutrient content depending on the timing of the treatment [13].

In potato production, foliar application of OSA significantly increased the content of vitamin C and starch in tubers [35,36,37]. A similar effect was obtained using GMC [39].

Foliar application of CS to winter rapeseed had no significant effect on fat, protein, and acid detergent fiber content, while significantly increasing detergent-neutral fiber content in winter rapeseed seeds [15]. The combination of soil fertilization with fertilizer containing Si, Ca, and Mg and foliar application of CS had an ambiguous effect on moisture, but a significant effect on 1000-kernel weight and number of kernels per cob [14]. In other studies, OSA application had no significant effect on grain moisture, 1000-kernel weight, or number of kernels per cob [40].

### 3.4. Biological Sugar Yield and Pure Sugar Yield

In our study, each form of silicon significantly increased biological and technological sugar yields compared to the control combination, with the greatest increase achieved after application of CS. CS had the most favorable effect on NDVI and LAI, resulting in high root yield (similar to that achieved after SM application) and the highest sugar content in the roots of all the treatments. It is difficult to explain why CS had the most beneficial effect on sugar beet yield. It may also have been due to the influence of Ca. The results of studies to date on the foliar application of various forms of silicon are inconclusive. In previous studies, foliar application of PS resulted in significantly higher biological sugar yield than SM, and similar to CS and OSA. Foliar application of PS and OSA resulted in significantly higher technological sugar yield than SM, and similar to CS [13]. Beneficial effects on biological sugar yield and technological sugar yield were also noted using SSN [34], CS, OSA, MS [12], and PS [16].

The latest application date contributed to a significant increase in biological sugar yield and pure sugar yield. In previous studies, the application date of various forms of silicon did not significantly affect biological sugar yield [13].

Foliar application of CS in winter rapeseed had no significant effect on fat yield in winter rapeseed [15].

## 4. Materials and Methods

### 4.1. Soil and Climatic Conditions

In 2017–2019, a field experiment with sugar beet was conducted in Sahryń, Poland (50°40′42″ N, 23°47′35″ E). The experiment was conducted on Calcic Chernozem [42]. Soil samples were collected after harvesting the previous crop from the 0–30 cm layer. Soil samples were analyzed at the District Chemical and Agricultural Stations in Warszawa-Wesoła. Soil pH (pH_KCl_) was determined potentiometrically in 1 M KCl [43], as well as the content of soil organic carbon (Corg) [44], nitrate nitrogen (N-NO_3_) and ammonium nitrogen (N-NH_4_) [45], available macroelements (P [46], K [47] and Mg [48]) and available microelements (B [49], Cu [50], Fe [51], Mn [52] and Zn [53]) (Table 14).

**Figure 3:**
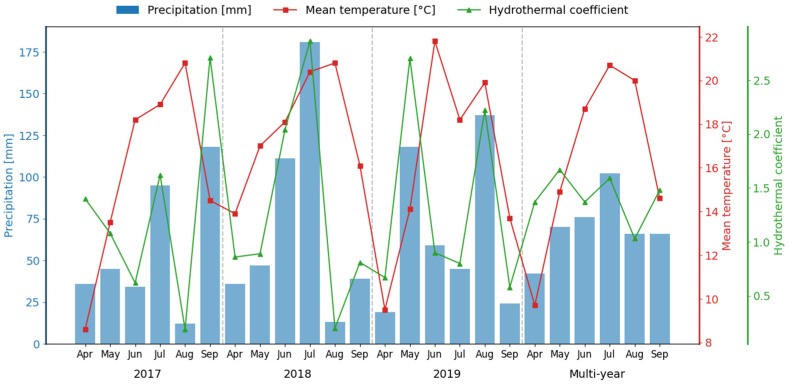
Weather conditions during the growing season of sugar beet (2017–2019). Precipitation 1991–2025; temperature: 2002–2025. Source: own study based on data from Strzyżów Sugar Factory [54] and https://www.edwin.gov.pl/dane-agrometeorologiczne, accessed on 10 March 2026 [55].

Precipitation during the April–September period ranged from 340 mm in 2017 to 427 mm a year later (Figure 3).

### 4.2. Crop Management

Sugar beet was cultivated in a no-till system after winter rapeseed. After winter rapeseed harvest, post-harvest tillage was performed, which was repeated as weeds and volunteer rapeseed germinated. In the second week of October, Polifoska 6 (N—60 g kg^−1^, P—87.2 g kg^−1^, K—249 g kg^−1^, S—28 g kg^−1^) was applied at a rate of 400 kg ha^−1^ and potassium salt (K—498 g kg^−1^) at a rate of 300 kg ha^−1^, for a total of 24 kg ha^−1^ N, 35 kg ha^−1^ P, 249 kg ha^−1^ K, and 11 kg ha^−1^ S. These fertilizers were mixed with the soil by deeper cultivation. In spring, Saletrzak 27 Standard with boron (N—270 g kg^−1^, Ca—14.3 g kg^−1^, Mg—24.1 g kg^−1^, B—2 g kg^−1^) was sown at a dose of 500 kg ha^−1^, providing 135 kg ha^−1^ N, 7 kg ha^−1^ Ca, 12 kg ha^−1^ Mg, and 1 kg ha^−1^ B. The fertilizer was mixed with the soil using a cultivator. The treatment was repeated just before sowing sugar beet. Foliar application of boron (B) was performed at the 6-leaf stage of beet root—BBCH 16 (Biologische Bundesanstalt, Bundessortenamt und Chemical Industry growth scale [56])—and 14 days later, foliar application of boron (B) was performed using Adob Bor fertilizer (N—78 g dm^−3^, B—150 g dm^−3^) at a dose of 2 dm^3^ ha^−1^ in each treatment. A total of 0.3 kg ha^−1^ N and 0.6 kg ha^−1^ B were supplied.

Sugar beet cultivar Toleranza KWS (KWS) was sown, depending on the year of the study, from 29 March to 10 April. This is a diploid variety of normal type (N), entered into the National Register in 2015. It is characterized by high root yield, good technological quality, and very high sugar yield [57]. The cultivar was sown at row spacing 45 cm, row distance 18 cm, and sowing depth 2–2.5 cm. In 2017–2019, the plant density at harvest was 87.1 thousand plants ha^−1^ and ranged from 82.2 thousand plants ha^−1^ in 2018 to 91.0 thousand plants ha^−1^ the previous year. Pest control was conducted in accordance with the recommendations of the Integrated Sugar and Fodder Beet Management Methodology for Advisors [58]. Sugar beet harvesting took place, depending on the year, from 27 September to 11 October. The length of the growing season ranged from 179 to 193 days.

### 4.3. Methodology

The experiment assessed the effect of three factors: silicon form (A), application date (B), and application dose (C) (Table 15 and Table 16). Application was performed depending on the combination: at the 6-leaf stage of sugar beet (B1), 7 days later (B2), and 14 days later (B3). Product rates were based on the manufacturers’ recommendations: Barrier Si-Ca (0.5 and 1 dm^3^ ha^−1^), ProHorti Si Max (1 and 2 dm^3^ ha^−1^), and TopSi (0.3 and 0.6 dm^3^ ha^−1^). The control combinations were treated with water on three dates. The water rate in each application was 250 dm^3^ ha^−1^. The working liquid was prepared immediately before foliar application. Spraying was performed with an Apollo trailed sprayer (Krukowiak, Brześć Kujawski, Poland) equipped with TeeJet flat spray tips, with working pressure 0.3 MPa.

The experiment was designed using the randomized block method. There were 18 experimental combinations + 3 controls, 4 replicates, and a total of 84 plots. Each plot consisted of 6 rows, 16 m long and 2.7 m wide. The area of each plot was 43.2 m^2^. The three middle rows (21.6 m^2^) were designated for harvesting.

In 2017, treatments were performed on 28 May, 4 June, and 11 June. In 2018, treatments were performed on 27 May, 3 June, and 10 June, respectively, and in 2019, on 26 May, 2 June, and 9 June, respectively.

On the day of each application and 7 days after the last treatment, the following plant physiological parameters were measured:

(1) Normalized Difference Vegetation Index (NDVI);
(2) Leaf Area Index (LAI);
(3) Photosynthetically active radiation (PAR) absorption.

In 2017, physiological parameters were measured on 28 May, 4, 11 June, and 18 June, in 2018, 27 May, 3, 10, and 17 June, and in 2019, 26 May, 2, 9, and 16 June.

NDVI measurements were taken with a GreenSeeker device (Trimble, Westminster, CO, USA). Leaf Area Index (LAI) and photosynthetically active radiation (PAR) were measured above and below the canopy with an AccuPar probe (Decagon, San Francisco, CA, USA). PAR absorption was calculated as the difference in PAR intensity above and below the canopy/PAR intensity above the canopy × 100%. Measurements were taken on 10 plants in the 3 middle rows of each plot. A total of 40 measurements were taken on each experimental combination at each date.

During harvest, the plants were topped by hand on the 3 middle rows, and the leaves were weighed. The roots were then counted, dug up, and weighed. During the harvest, each plot was collected in accordance with the Polish Standard [59]. Root technological quality was assessed on the automatic Venema technological line in Straszków (Kutno Sugar Beet Breeding Company, Straszków, Poland)—sugar content polarimetrically, α-amino nitrogen, K, and Na—by photoelectric flame photometry. Measurements performed in the experiments include:

1. Normalized Difference Vegetation Index (NDVI);
2. Leaf Area Index (LAI);
3. Photosynthetically active radiation (PAR) absorption;
4. Root yield (t ha^−1^);
5. Sugar content in roots (%);
6. α-amino nitrogen content in the roots (mmol kg^−1^);
7. Potassium (K) content in the roots (mmol kg^−1^);
8. Sodium (Na) content in the roots (mmol kg^−1^);
9. Alkalinity coefficient = [K content (mmol kg^−1^) + Na content (mmol kg^−1^)]/α-amino N content (mmol kg^−1^) [60];
10. Biological sugar yield (t ha^−1^) = product of root yield (t ha^−1^) and sugar content in roots (%);
11. Pure sugar yield (t ha^−1^) = root yield (t ha^−1^) × [content of sugar (%) − sugar yield losses (%)] [41];
12. Sugar yield losses (%) = standard molasses losses (%) + 0.6 (%);
13. Standard molasses losses (%) = 0.012 × (K + Na) + 0.024 (α-amino nitrogen) + 0.48.

The content of K, Na, and α-amino nitrogen are given in mmol kg^−1^ of pulp.

The experimental results were statistically analyzed using a multi-way analysis of variance (ANOVA), in which study years were treated as a fixed factor due to their limited number. The model included the effects of year, silicon form, application timing, and product dose, as well as their interactions. Mean comparisons were performed using Tukey’s HSD test at *p* = 0.05. A significance level of *p* = 0.05 was adopted for comparison of means. Based on this, homogeneous groups of means were identified, which are designated by consecutive letters of the alphabet. To characterize individual traits, minimum and maximum values, standard deviation, and coefficient of variation were calculated. The correlation between physiological parameters and yield traits was assessed based on Pearson’s simple correlation coefficients. Significance of correlations was assessed at *p* ≤ 0.05 and *p* ≤ 0.01. Results are presented in tables and figures.

Principal component analysis (PCA) based on the correlation matrix was applied for evaluation of relationships between studied variables and multivariate differences between the variants.

Analyses were performed using Statistica 13.3 (TIBCO Software Inc., Palo Alto, CA, USA).

## 5. Conclusions

Although orthosilicic acid is considered the only form of silicon taken up by plants, our research has shown that foliar application of silicon in forms other than stabilized orthosilicic acid (calcium silicate, sodium metasilicate, and micronized silica) is effective. These forms are significantly cheaper than stabilized orthosilicic acid, which may reduce production costs. The most effective results were obtained using calcium silicate. To achieve maximum results, the application should be performed when plants have a sufficiently developed leaf area, at least seven days after reaching the six-leaf stage. Increasing the dose of the products is not recommended, as it does not significantly improve sugar beet yield. Further research is recommended in multiple locations with diverse soil and climatic conditions.

## Figures and Tables

**Table 1:** *p* values based on analysis of variance for assessed physiological parameters (2017–2019).

	Measurement 1			Measurement 2			Measurement 3			Measurement 4		
	NDVI	LAI	PAR Absorption	NDVI	LAI	PAR Absorption	NDVI	LAI	PAR Absorption	NDVI	LAI	PAR Absorption
Y	<0.05	<0.05	<0.05	<0.05	<0.05	<0.05	<0.05	<0.05	<0.05	<0.05	<0.05	<0.05
A	<0.05	<0.05	<0.05	<0.05	<0.05	<0.05	<0.05	<0.05	<0.05	<0.05	<0.05	<0.05
B	0.992	0.197	0.393	0.646	<0.05	<0.05	<0.05	<0.05	0.243	<0.05	<0.05	<0.05
C	0.825	0.958	0.708	0.223	0.771	0.114	<0.05	0.297	0.494	0.100	0.068	0.137
Y × A	<0.05	<0.05	<0.05	<0.05	<0.05	<0.05	<0.05	<0.05	<0.05	<0.05	<0.05	<0.05
Y × B	<0.05	<0.05	<0.05	0.203	<0.05	<0.05	<0.05	<0.05	<0.05	<0.05	<0.05	<0.05
A × B	<0.05	<0.05	<0.05	<0.05	<0.05	<0.05	<0.05	<0.05	<0.05	<0.05	<0.05	<0.05
Y × C	0.971	0.983	0.936	0.566	0.246	0.186	<0.05	<0.05	<0.05	0.419	0.189	<0.05
A × C	0.987	1.000	0.742	<0.05	<0.05	<0.05	<0.05	0.670	0.688	<0.05	0.754	0.053
B × C	0.882	0.976	0.923	<0.05	<0.05	0.173	<0.05	0.954	0.429	0.055	0.829	<0.05
Y × A × B	<0.05	<0.05	<0.05	<0.05	<0.05	<0.05	<0.05	<0.05	<0.05	<0.05	<0.05	<0.05
Y × A × C	1.000	1.000	0.998	0.826	0.684	0.812	0.150	0.000	0.058	0.100	<0.05	<0.05
Y × B × C	0.993	0.999	0.971	<0.05	<0.05	<0.05	<0.05	0.105	0.145	<0.05	0.328	<0.05
A × B × C	0.998	1.000	0.999	<0.05	<0.05	<0.05	0.427	<0.05	<0.05	0.107	0.182	<0.05
Y × A × B × C	1.000	1.000	0.999	<0.05	0.225	0.679	0.486	<0.05	0.075	0.195	<0.05	<0.05

Y—study year; A—silicon form; B—application date; C—dose; 1st date—BBCH 16; 2nd date—7 days later; 3rd date—14 days later; 4th date—21 days later.

**Table 2:** Characteristics of statistical variability of physiological parameters of sugar beet (2017–2019).

Term of Measurement	Physiological Parameter	Mean	Minimum	Maximum	Standard Deviation	Coefficient of Variation, %
1.	NDVI	0.28	0.16	0.54	0.05	19.37
	LAI	0.25	0.01	1.09	0.12	47.05
	PAR absorption	16.11	1.00	57.40	6.75	41.88
2.	NDVI	0.39	0.15	0.79	0.14	35.82
	LAI	0.97	0.10	2.69	0.38	39.70
	PAR absorption	44.62	9.53	85.31	11.60	25.99
3.	NDVI	0.61	0.24	0.83	0.11	17.60
	LAI	1.89	0.40	4.61	0.76	40.27
	PAR absorption	64.64	21.70	96.90	16.02	24.79
4.	NDVI	0.76	0.43	0.88	0.07	9.07
	LAI	2.53	0.90	5.48	0.65	25.57
	PAR absorption	77.71	20.60	97.30	9.59	12.34

1st term—BBCH 16; 2nd term—7 days later; 3rd term—14 days later; 4th term—21 days later.

**Table 3:** Correlations of sugar beet physiological parameters with its yield (2017–2019); n = 252.

Term of Measurement	Physiological Parameter	Root Yield, t ha^−1^	Sugar Content in Roots, %	Biological Sugar Yield, t ha^−1^	Pure Sugar Yield, t ha^−1^
1.	NDVI	−0.035	0.032	−0.048	−0.045
	LAI	0.475	−0.295	0.422	0.383
	PAR absorption	0.349	−0.235	0.303	0.270
2.	NDVI	0.624	−0.655	0.449	0.383
	LAI	0.079	−0.086	0.040	0.032
	PAR absorption	0.131	0.171	0.223	0.241
3.	NDVI	−0.031	0.497	0.167	0.213
	LAI	0.587	−0.667	0.388	0.314
	PAR absorption	0.639	−0.662	0.461	0.386
4.	NDVI	0.677	−0.713	0.492	0.415
	LAI	0.070	−0.093	0.032	0.013
	PAR absorption	−0.106	−0.024	−0.147	−0.158

1st term—BBCH 16; 2nd term—7 days later; 3rd term—14 days later; 4th term—21 days later.

**Table 4:** *p* values based on analysis of variance for the evaluated yield parameters (2017–2019).

Effect	Root Yield	Content in Roots				Alkalinity Coefficient	Biological Sugar Yield	Pure Sugar Yield
		Sugar	K	Na	N α-Amino			
Y	<0.05	<0.05	<0.05	<0.05	<0.05	<0.05	<0.05	<0.05
A	<0.05	<0.05	<0.05	<0.05	0.061	<0.05	<0.05	<0.05
B	<0.05	0.064	0.103	0.067	<0.05	0.061	<0.05	0.060
C	0.712	<0.05	0.140	<0.05	0.603	0.930	0.324	0.267
Y × A	<0.05	<0.05	<0.05	<0.05	<0.05	<0.05	<0.05	<0.05
Y × B	<0.05	<0.05	<0.05	0.101	<0.05	0.412	<0.05	<0.05
A × B	0.072	<0.05	<0.05	<0.05	<0.05	0.399	<0.05	<0.05
Y × C	0.178	<0.05	<0.05	<0.05	<0.05	0.778	0.390	0.346
A × C	0.649	<0.05	0.056	<0.05	<0.05	<0.05	0.319	0.284
B × C	0.109	0.578	0.201	<0.05	<0.05	0.098	0.118	0.139
Y × A × B	<0.05	<0.05	<0.05	<0.05	<0.05	<0.05	<0.05	<0.05
Y × A × C	0.128	<0.05	<0.05	<0.05	<0.05	<0.05	0.075	0.099
Y × B × C	0.361	0.487	0.423	<0.05	0.353	0.989	0.387	0.374
A × B × C	0.600	0.485	<0.05	<0.05	<0.05	<0.05	0.678	0.621
Y × A × B × C	0.511	<0.05	0.053	<0.05	0.278	0.248	0.423	0.405

Y—years of research; A—form of silicon; B—application date; C—dose; n = 252.

**Table 5:** Sugar beet root yield depending on the form of silicon, date of foliar application and dose (2017–2019), t ha^−1^.

Silicon Form ^1^ (A)	Term of Application (B)						Mean
	B1		B2		B3		
	Dose (C)		Dose (C)		Dose (C)		
	C1	C2	C1	C2	C1	C2	
Control	80.29 ± 11.13 a		81.28 ± 12.74 ab		80.65 ± 12.69 a		80.72 ± 11.82 A
CS (A1)	88.35 ± 11.36 cde ^2^	85.41 ± 13.25 abc	92.69 ± 11.38 ef	95.37 ± 11.38 fg	99.03 ± 11.59 g	92.94 ± 14.32 efg	92.30 ± 12.65 C
SM (A2)	89.49 ± 15.51 cdef	88.62 ± 15.36 cde	89.14 ± 16.32 cdef	91.85 ± 15.34 def	92.13 ± 17.22 def	89.15 ± 20.06 cdef	90.06 ± 16.18 C
MS (A3)	83.90 ± 9.15 abc	89.38 ± 13.08 cdef	86.00 ± 14.23 abcd	86.96 ± 15.08 bcde	88.30± 10.61 cde	85.39 ± 17.17 abc	86.65 ± 13.14 B
Mean	85.71 ± 12.76 A		88.07 ± 14.11 B		88.53 ± 15.48 B		
	C1			C2			
Mean	87.60 ± 13.63 *A*			87.26 ± 14.73 *A*			

^1^ Control—without Si fertilization, A1—CS: silicon in the form of calcium silicate (Barrier Si-Ca); A2—SM: silicon in the form of sodium metasilicate (ProHorti Si Max); A3—MS: silicon in the form of micronized silica (TopSi); ^2^ the same lowercase letters indicate no significant differences between form, date, and dose combinations, while the same uppercase letters indicate no significant differences between form means (last column), between term means (row comparisons) or between dose means (capital letters in italics). All comparisons of means are at *p* = 0.05.

**Table 6:** Sugar content in sugar beet roots depending on the form of silicon, date of foliar application and dose (2017–2019), %.

Silicon Form ^1^ (A)	Term of Application (B)						Mean
	B1		B2		B3		
	Dose (C)		Dose (C)		Dose (C)		
	C1	C2	C1	C2	C1	C2	
Control	17.69 ± 1.08 fgh ^2^		17.61 ± 1.02 cdefgh		17.58 ± 1.04 cdefg		17.63 ± 1.01 C
CS (A1)	17.76 ± 1.18 ghi	17.84 ± 1.02 hi	18.08 ± 0.94 j	17.63 ± 1.05 defgh	17.92 ± 0.47 ij	17.65 ± 1.03 efgh	17.81 ± 0.95 D
SM (A2)	17.18 ± 1.41 ab	17.13 ± 1.16 a	17.10 ± 1.26 a	17.05 ± 1.46 a	17.52 ± 1.14 cdefg	17.55 ± 0.94 cdefg	17.25 ± 1.21 A
MS (A3)	17.63 ± 0.88 cdefgh	17.52 ± 1.07 cdef	17.41 ± 1.05 bcd	17.42 ± 0.79 cde	17.40 ± 0.82 bc	17.49 ± 1.18 cdef	17.48 ± 0.95 B
Mean	17.55 ± 1.11 AB		17.49 ± 1.09 A		17.59 ± 0.96 B		
	C1			C2			
Mean	17.51 ± 1.07 *A*			17.58± 1.03 *B*			

^1^ as in Table 5; ^2^ as in Table 5.

**Table 7:** Content of α-amino nitrogen in sugar beet roots depending on the form of silicon, date of foliar application and dose (2017–2019), mmol kg^−1^.

Silicon Form ^1^ (A)	Term of Application (B)						Mean
	B1		B2		B3		
	Dose (C)		Dose (C)		Dose (C)		
	C1	C2	C1	C2	C1	C2	
Control	24.02 ± 6.50 bcd ^2^		24.05 ± 6.42 bcd		24.22 ± 6.05 bcd		24.10 ± 6.10 A
CS (A1)	24.22 ± 3.90 bcd	23.18 ± 2.14 b	23.25 ± 4.66 b	25.52 ± 3.63 def	27.55 ± 6.66 g	26.10 ± 5.11 efg	24.97 ± 4.68 B
SM (A2)	23.75 ± 6.21 bc	26.75 ± 7.68 fg	23.85 ± 5.99 bcd	23.98 ± 8.28 bcd	24.10 ± 6.39 bcd	26.07 ± 7.89 efg	24.75 ± 6.98 AB
MS (A3)	26.10 ± 5.98 efg	21.47 ± 6.06 a	23.62 ± 4.11 bc	25.47 ± 8.08 def	24.98 ± 5.79 cde	24.43 ± 7.76 bcde	24.34 ± 6.38 AB
Mean	24.19 ± 5.84 A		24.22 ± 5.98 A		25.21 ± 6.39 B		
	C1			C2			
Mean	24.60 ± 6.46 *A*			24.47 ± 5.69 *A*			

^1^ as in Table 5; ^2^ as in Table 5.

**Table 8:** K content in sugar beet roots depending on the form of silicon, date of foliar application and dose (2017–2019), mmol kg^−1^.

Silicon Form ^1^ (A)	Term of Application (B)						Mean
	B1		B2		B3		
	Dose (C)		Dose (C)		Dose (C)		
	C1	C2	C1	C2	C1	C2	
Control	32.57 ± 2.94 a ^2^		32.73 ± 2.81 a		32.43 ± 3.09 a		32.58 ± 2.85 A
CS (A1)	33.77 ± 3.77 abcd	34.40 ± 4.07 bcde	35.55 ± 2.63 efg	33.83 ± 1.73 abcd	37.00 ± 2.98 g	36.22 ± 2.93 fg	35.13 ± 3.24 C
SM (A2)	33.42 ± 5.00 abcd	34.37 ± 3.61 bcde	32.78 ± 5.08 a	34.78 ± 3.62 cdef	34.47 ± 5.94 bcde	32.94 ± 3.84 ab	33.79 ± 4.50 B
MS (A3)	34.95 ± 2.85 def	32.58 ± 2.66 a	34.72 ± 2.31 cdef	34.74 ± 1.93 cdef	34.58 ± 4.88 cde	33.27 ± 4.75 abc	34.14 ± 3.43 B
Mean	33.58 ± 3.54 A		33.98 ± 3.11 AB		34.17 ± 4.25 B		
	C1			C2			
Mean	33.74 ± 3.34 *A*			34.08 ± 3.95 *A*			

^1^ as in Table 5; ^2^ as in Table 5.

**Table 9:** Na content in sugar beet roots depending on the form of silicon, date of foliar application and dose (2017–2019), mmol kg^−1^.

Silicon Form ^1^ (A)	Term of Application (B)						Mean
	B1		B2		B3		
	Dose (C)		Dose (C)		Dose (C)		
	C1	C2	C1	C2	C1	C2	
Control	2.78 ± 0.91 bc ^2^		2.90 ± 0.97 bcd		2.88 ± 0.87 bcd		2.85 ± 0.88 B
CS (A1)	2.37 ± 0.20 a	2.60 ± 0.62 ab	2.72 ± 0.64 bc	2.58 ± 0.26 ab	3.03 ± 0.95 cde	2.78 ± 0.73 bc	2.68 ± 0.64 A
SM (A2)	3.40 ± 1.31 fg	3.82 ± 1.54 hi	3.45 ± 1.37 fg	4.00 ± 1.59 ij	3.65 ± 1.44 gh	3.17 ± 0.90 dfg	3.58 ± 1.36 C
MS (A3)	3.32 ± 0.82 efg	4.20 ± 1.73 jk	3.83 ± 1.17 hi	3.52 ± 0.62 gh	3.52 ± 1.24 gh	4.52 ± 2.33 k	3.82 ± 1.45 D
Mean	3.16 ± 1.22 A		3.24 ± 1.11 AB		3.30 ± 1.33 B		
	C1			C2			
Mean	3.31 ± 1.34 *B*			3.15 ± 1.09 *A*			

^1^ as in Table 5; ^2^ as in Table 5.

**Table 10:** Alkalinity coefficient depending on the form of silicon, the date of foliar application and the dose (2017–2019).

Silicon Form ^1^ (A)	Term of Application (B)						Mean
	B1		B2		B3		
	Dose (C)		Dose (C)		Dose (C)		
	C1	C2	C1	C2	C1	C2	
Control	1.58 ± 0.48 abcdef ^2^		1.58 ± 0.44 abcdef		1.58 ± 0.38 abcde		1.57 ± 0.42 AB
CS (A1)	1.51 ± 0.19 abc	1.61 ± 0.25 bcdef	1.71 ± 0.36 fgh	1.46 ± 0.26 a	1.56 ± 0.48 abcde	1.54 ± 0.28 abcde	1.56 ± 0.32 A
SM (A2)	1.64 ± 0.41 cdefg	1.50 ± 0.27 ab	1.62 ± 0.50 bcdef	1.76 ± 0.47 gh	1.67 ± 0.43 efg	1.53 ± 0.52 abc	1.62 ± 0.43 BC
MS (A3)	1.53 ± 0.35 abcd	1.80 ± 0.37 h	1.66 ± 0.20 defg	1.63 ± 0.46 bcdefg	1.57 ± 0.24 abcde	1.62 ± 0.26 bcdefg	1.64 ± 0.32 C
Mean	1.60 ± 0.36 AB		1.63 ± 0.40 B		1.57 ± 0.37 A		
	C1			C2			
Mean	1.60 ± 0.38 *A*			1.60 ± 0.38 *A*			

^1^ as in Table 5; ^2^ as in Table 5.

**Table 11:** Biological sugar yield depending on the form of silicon, the date of foliar application and the dose (2017–2019), t ha^−1^.

Silicon Form ^1^ (A)	Term of Application (B)						Mean
	B1		B2		B3		
	Dose (C)		Dose (C)		Dose (C)		
	C1	C2	C1	C2	C1	C2	
Control	14.13 ± 1.67 ab ^2^		14.22 ± 1.69 ab		14.09 ± 1.78 a		14.14 ± 1.66 A
CS (A1)	15.67 ± 1.34 cdef	15.06 ± 1.80 abcd	16.73 ± 1.96 fgh	16.85 ± 2.44 gh	17.72 ± 1.86 h	16.31 ± 2.03 efg	16.39 ± 2.05 C
SM (A2)	15.46 ± 3.31 cde	15.12 ± 2.42 abcd	15.18 ± 2.67 bcd	15.56 ± 2.28 cde	16.00 ± 2.34 defg	15.51 ± 3.00 cde	15.47 ± 2.61 B
MS (A3)	14.74 ± 1.27 abc	15.56 ± 1.59 cde	14.85 ± 1.65 abc	15.05 ± 2.01 abcd	15.30 ± 1.39 cde	14.78 ± 2.17 abc	15.05 ± 1.68 B
Mean	14.98 ± 2.00 A		15.33 ± 2.21 AB		15.47 ± 2.31 B		
	C1			C2			
Mean	15.18 ± 2.19 *A*			15.34 ± 2.18 *A*			

^1^ as in Table 5; ^2^ as in Table 5.

**Table 12:** Pure sugar yield of sugar depending on the form of silicon, the date of foliar application and the dose (2017–2019), t ha^−1^.

Silicon Form ^1^ (A)	Term of Application (B)						Mean
	B1		B2		B3		
	Dose (C)		Dose (C)		Dose (C)		
	C1	C2	C1	C2	C1	C2	
Control	12.45 ± 1.37 a ^2^		12.51 ± 1.33 a		12.39 ± 1.44 a		12.45 ± 1.34 A
CS (A1)	13.81 ± 1.08 cde	13.28 ± 1.56 abcd	14.79 ± 1.77 fg	14.82 ± 2.23 fg	15.51± 1.58 g	14.27 ± 1.68 ef	14.41 ± 1.78 C
SM (A2)	13.58 ± 2.98 cde	13.17 ± 2.01 abcd	13.30 ± 2.29 abcd	13.58 ± 1.88 cde	14.02± 1.90 def	13.58 ± 2.44 cde	13.54 ± 2.22 B
MS (A3)	12.92 ± 1.07 abc	13.72 ± 1.24 cde	13.02 ± 1.29 abc	13.16 ± 1.56 abcd	13.41± 1.19 bcde	12.94 ± 1.67 abc	13.19 ± 1.34 B
Mean	13.17 ± 1.71 A		13.46 ± 1.89 AB		13.56 ± 1.91 B		
	C1			C2			
Mean	13.32 ± 1.82 *A*			13.48 ± 1.87 *A*			

^1^ as in Table 5; ^2^ as in Table 5.

**Table 13:** Descriptive statistics for all experiments with sugar beet (2017–2019).

Trait	Mean	Minimum	Maximum	Standard Deviation (SD)	Coefficient of Variation (CV), %
Root yield, t ha^−1^	87.43	57.36	112.78	14.17	16.20
Content of sugar in the roots, %	17.54	14.57	19.47	1.05	5.99
Content of α-amino nitrogen in the roots, mmol kg^−1^	24.54	13.60	37.40	6.07	24.75
K content in the roots, mmol kg^−1^	33.91	27.10	46.40	3.66	10.78
Na content in the roots, mmol kg^−1^	3.23	1.40	8.90	1.22	37.83
Alkalinity coefficient	1.60	0.92	2.52	0.38	23.58
Biological sugar yield, t ha^−1^	15.26	9.33	20.01	2.18	14.30
Pure sugar yield, t ha^−1^	13.40	7.99	17.63	1.84	13.74

**Table 14:** Soil conditions before establishing the experiment (2016–2018).

pH_KCl_	Corg %	mg kg^−1^		mg kg^−1^							
		N-NO_3_	N-NH_4_	P	K	Mg	B	Cu	Fe	Mn	Zn
2016											
7.4	2.10	80.5	4.98	65.0	100	69	2.60	6.6	500	160	6.5
2017											
7.5	2.40	46.0	2.68	82.4	82	71	2.20	7.0	510	150	6.9
2018											
7.3	2.70	28.3	3.01	79.1	123	79	3.06	8.2	610	159	7.30

**Table 15:** Chemical composition of the products used in the experiment.

Product	Content in 1 dm^3^
Barrier Si-Ca	Si (in the form of calcium silicate, pH 9.5–11.0)—158 g; Ca—207 g.
ProHorti Si Max	Si (in the form of sodium metasilicate)—58 g; N—62 g; P—16 g; K—71 g; plant hormones (auxins, cytokinins, gibberellins), polysaccharides (alginates, mannitol), as well as vitamins (A, B, C, E), amino acids and polyphenols from marine algae *Ecklonia maxima*.
TopSi	Si (in the form of micronized silica)—99 g; N—9 g; B—17 g; Fe—28 g; Ti—30 g.

Source: manufacturers’ labels.

**Table 16:** Variants used in the experiment.

Form of Silicon (A)	Application Date (B)	Product Dose (C)	Quantity of Ingredients Supplied, g ha^−1^
Control	6-leaf stage of sugar beet—BBCH 16 (B1)	–	–
	7 days later (B2)	–	–
	14 days later (B3)	–	–
CS (A1)	B1	Full (C1) Double (C2)	Si—79; Ca—104 Si—158; Ca—207 Si—79; Ca—104 Si—158; Ca—207 Si—79; Ca—104 Si—158; Ca—207
	B2	C1 C2	
	B3	C1 C2	
SM (A2)	B1	C1 C2	Si—58; N—62; P—16; K—71 Si—116; N—124; P—32; K—142 Si—58; N—62; P—16; K—71 Si—116; N—124; P—32; K—142 Si—58; N—62; P—16; K—71 Si—116; N—124; P—32; K—142
	B2	C1 C2	
	B3	C1 C2	
MS (A3)	B1	C1 C2	Si—25; N—2; B—5; Fe—7; Ti—8 Si—50; N—5; B—9; Fe—14; Ti—15 Si—25; N—2; B—5; Fe—7; Ti—8 Si—50; N—5; B—9; Fe—14; Ti—15 Si—25; N—2; B—5; Fe—7; Ti—8 Si—50; N—5; B—9; Fe—14; Ti—15
	B2	C1 C2	
	B3	C1 C2	

## Data Availability

Data are available upon request from the corresponding author.

## Supplementary Materials

The following supporting information can be downloaded at https://www.mdpi.com/article/10.3390/plants15101449/s1. Figure S1: Changes in NDVI (charts 1d), LAI (charts 2d) and PAR absorption (charts 3d) depending on product dose (C1—full, C2—double). Vertical lines indicate standard deviations. The same letters indicate no significant differences at *p* = 0.05.

## Abbreviations

The following abbreviations are used in this manuscript:

CS	calcium silicate
GMC	ground marine calcite
MS	micronized silica
OSA	orthosilicic acid
PS	potassium silicate
SM	sodium metasilicate
SSN	stabilized silica nanoparticles
PAR	photosynthetically active radiation
LAI	Leaf Area Index
NDVI	Normalized Difference Vegetation Index
SPAD	Soil–Plant Analysis Development
