# Supplementary material for: Do Forms of Silicon Other than Orthosilicic Acid, Application Date and Dose Have a Beneficial Effect on Sugar Beet Yield?
Source: Plants (Basel). 2026 May 9;15(10):1449. doi: 10.3390/plants15101449 (PMC13211158; doi:10.3390/plants15101449)
Supplement: Supplementary file 1 [file plants-15-01449-s001.zip › plants-4284533-supplementary.pdf]

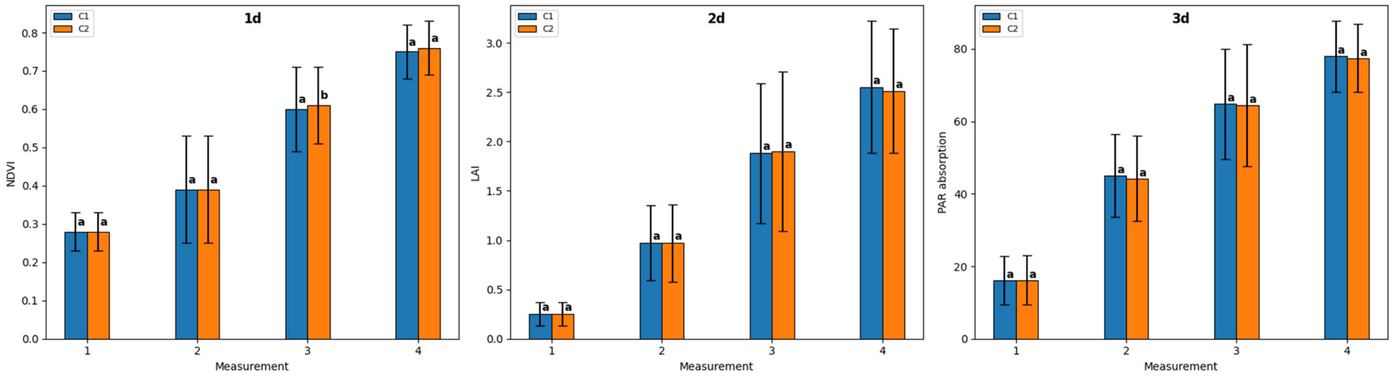

Figure S1. Changes in NDVI (charts 1d), LAI (charts 2d) and PAR absorption (charts 3d) depending on product dose (C1—full, C2—double). Vertical lines indicate standard deviations. The same letters indicate no significant differences at $p = 0.05$.
